# Many-Exciton Quantum Dynamics in a Ruddlesden–Popper Tin Iodide

**DOI:** 10.1021/acs.jpcc.3c04896

**Published:** 2023-10-25

**Authors:** Esteban Rojas-Gatjens, Hao Li, Alejandro Vega-Flick, Daniele Cortecchia, Annamaria Petrozza, Eric R. Bittner, Ajay Ram Srimath Kandada, Carlos Silva-Acuña

**Affiliations:** † School of Chemistry and Biochemistry, Georgia Institute of Technology, Atlanta, Georgia, 30332, United States; ‡ School of Physics, Georgia Institute of Technology, Atlanta, Georgia, 30332, United States; § Department of Chemistry, University of Houston, Houston, Texas 77204, United States; ∥ Center for Nano Science and Technology@PoliMi, Istituto Italiano di Tecnologia, Milan 20133, Italy; ⊥ Center for Nonlinear Studies, Los Alamos National Laboratory, Los Alamos, New Mexico 87544, United States; # Department of Physics, Wake Forest University, Winston–Salem, North Carolina 27587, United States; ¶ Center for Functional Materials, Wake Forest University, Winston–Salem, North Carolina 27109, United States; ∇ School of Materials Science and Engineering, Georgia Institute of Technology, Atlanta, Georgia, 30332, United States

## Abstract

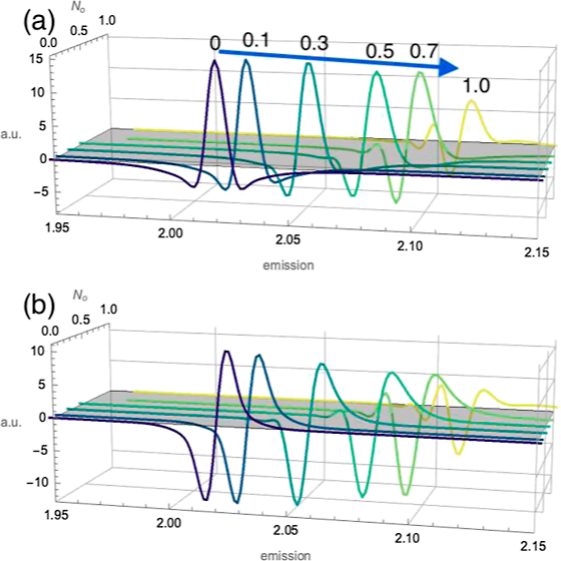

We present a study on the many-body exciton interactions in a Ruddlesden–Popper tin halide, namely, (PEA)_2_SnI_4_ (PEA = phenylethylammonium), using coherent two-dimensional electronic spectroscopy. The optical dephasing times of the third-order polarization observed in these systems are determined by exciton many-body interactions and lattice fluctuations. We investigate the excitation-induced dephasing (EID) and observe a significant reduction of the dephasing time with increasing excitation density as compared to its lead counterpart (PEA)_2_PbI_4_, which we have previously reported in a separate publication [*J. Chem. Phys.***2020**, 153, 164706]. Surprisingly, we find that the EID interaction parameter is four orders of magnitude higher in (PEA)_2_SnI_4_ than that in (PEA)_2_PbI_4_. This increase in the EID rate may be due to exciton localization arising from a more statically disordered lattice in the tin derivative. This is supported by the observation of multiple closely spaced exciton states and the broadening of the linewidth with increasing population time (spectral diffusion), which suggests a static disordered structure relative to the highly dynamic lead-halide. Additionally, we find that the exciton nonlinear coherent lineshape shows evidence of a biexcitonic state with low binding energy (<10 meV) not observed in the lead system. We model the lineshapes based on a stochastic scattering theory that accounts for the interaction with a nonstationary population of dark background excitations. Our study provides evidence of differences in the exciton quantum dynamics between tin- and lead-based Ruddlesden–Popper metal halides (RPMHs) and links them to the exciton–exciton interaction strength and the static disorder aspect of the crystalline structure.

## Introduction

Ruddlesden–Popper metal halides (RPMHs) are a unique class of semiconductors with a layered crystal structure, consisting of alternating metal-halide perovskite-like layers and long organic molecular moieties that separate them, forming a quasi-two-dimensional material. As a result of their structure, these materials host strongly bound excitons at ambient conditions,^1^ making them attractive for use in quantum optoelectronics applications such as photonic lasers^2−10^ and exciton-polariton coherent emitters.^11,12^ In these quantum technologies, the quantum dynamics of excitons are deterministic since both population and coherence relaxation times are profoundly influenced by many-body exciton Coulomb correlations. A highly relevant example of such a phenomenon is excitation-induced dephasing (EID). EID arises due to instantaneous incoherent elastic scattering between multiple excitons, leading to faster dephasing dynamics of the mesoscopic polarization than the low-density pure-dephasing limit, and can be the dominant dephasing pathway at sufficiently high exciton densities. This process, thus, strongly determines optical lineshapes and limits homogeneous line widths,^13−15^ which in turn govern the material’s optical properties. A fundamental aspect of exciton many-body interactions in RPMHs is their strongly polaronic character.^16−18^ We have suggested that the primary photoexcitations in RPMHs are exciton polarons, which are quasiparticles in which the anharmonic phonons and the Coulomb-bound electron–hole pairs are both integral components of the electronic eigenstates of the system. We have rationalized that the presence of distinct excitonic resonances in the optical spectra of RPMHs is due to a family of distinct exciton polarons with distinct lattice dressing.^17^ Notably, some researchers interpret the spectral structure as a vibronic progression,^19−23^ as opposed to our interpretation of distinct exciton polarons.^18^ In our previous study of (PEA)_2_PbI_4_, we observed that the EID signatures are different for each of the resonances. If they belong to the same electronic excited manifold, then the many-body scattering should not vary significantly. Instead, we find that the polaronic dressings of excitonics present significant yet distinct consequences on the many-body quantum dynamics in RPMHs as observed through EID.

Signatures of EID evidently manifest in the nonlinear coherent optical lineshapes.^24−26^ A noninteracting coherent population typically results in a characteristic absorptive line shape in the real part of the two-dimensional coherent spectrum, composed of a symmetric, positive feature along the antidiagonal. The imaginary component of the spectrum concurrently exhibits a dispersive lineshape that has a positive slope along the antidiagonal. More precisely, for a two-level system, the rephasing response can be written as^13,27^
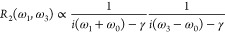
1where ω_0_ is the transition frequency and γ is the Lorentzian width due to homogeneous dephasing. The expected spectrum of the antidiagonal line of the two-dimensional spectrum, centered about ω_0_ and represented as ω_1_ = −ω_0_ + δ and ω_3_ = ω_0_ + δ, can be written as
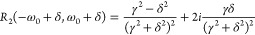
2

The above expression clearly shows that the real part of the rephasing signal must be an even function under reflection across the antidiagonal while the imaginary component must be an odd function. Moreover, it indicates that the imaginary part must have a positive slope at δ = 0 and the real part is positive at δ = 0. Notably, this is a mathematically exact result for the rephasing signal and should be expected for any isolated transition with homogeneous broadening of γ.

In the case of RPMHs, previous observations have shown that the coherent nonlinear response deviates from the expected lineshape.^28^ Instead, the real part of the spectrum appears to be an odd function with a positive slope along the antidiagonal line, while the imaginary component is clearly absorptive. Such a reversal of the expected lineshapes of the real and imaginary components is indicative of an additional phase shift in the nonlinear response function of the sample. This shift has been previously interpreted as a consequence of excitation-induced effects by Cundiff and co-workers, based on a phenomenological model.^24,29^ We generalized this model by explicitly considering stochastic scattering of optically accessible excitations with a background population of dark excitons at nonzero momenta that leads to the overall phase shift in the nonlinear response function.^28^

Our work establishes that scattering of the coherent exciton population with background excitations, a major portion of which is not optically inaccessible, is responsible for the observed relatively short coherence lifetimes in (PEA)_2_PbI_4_. These scattering processes manifest even at low excitation densities, but they reduce substantially within hundreds of femtoseconds, much faster than the overall loss of the background population, but within the expected timescale of polaronic interactions. We hypothesized that the EID dynamics are limited by the dynamic screening of the many-body Coulomb interactions by the ionic lattice.^28^ The critical role of the lattice and the exciton polaron hypothesis, although suggestive, has yet to be robustly substantiated. A convenient experimental parameter that may enable further exploration of this problem is through metal cation substitution without major disruption of the structural composition of the lattice. This can be achieved in the PEA derivatives in which the lead and tin counterparts have been shown to have similarities from a structural point of view.^30^

Although the effect of metal cation substitution from Pb to Sn and Ge has been extensively studied from a structural point of view,^30,31^ the changes it may induce in the electronic dynamics remain unclear. First, the choice of the metal ion is expected to modulate the strength of spin–orbit coupling,^32^ leading to consequences in the splitting energies between multiple conduction bands, composed of p-orbitals of the metal cation. Changes in the Rashba-Dresselhaus splitting at the band edge have also been predicted.^33^ Second, moving from the larger Pb ion to the smaller Sn (or Ge) can be expected to change the dynamic nature of the lattice due to enhanced organic–inorganic interactions, lattice polarizability, and consequently, the excitonic properties.^22,34−36^ Both of the above-stated consequences of the metal cation substitution can lead to pertinent changes in the many-body interactions and, consequently, the EID dynamics.

**Figure 1:**
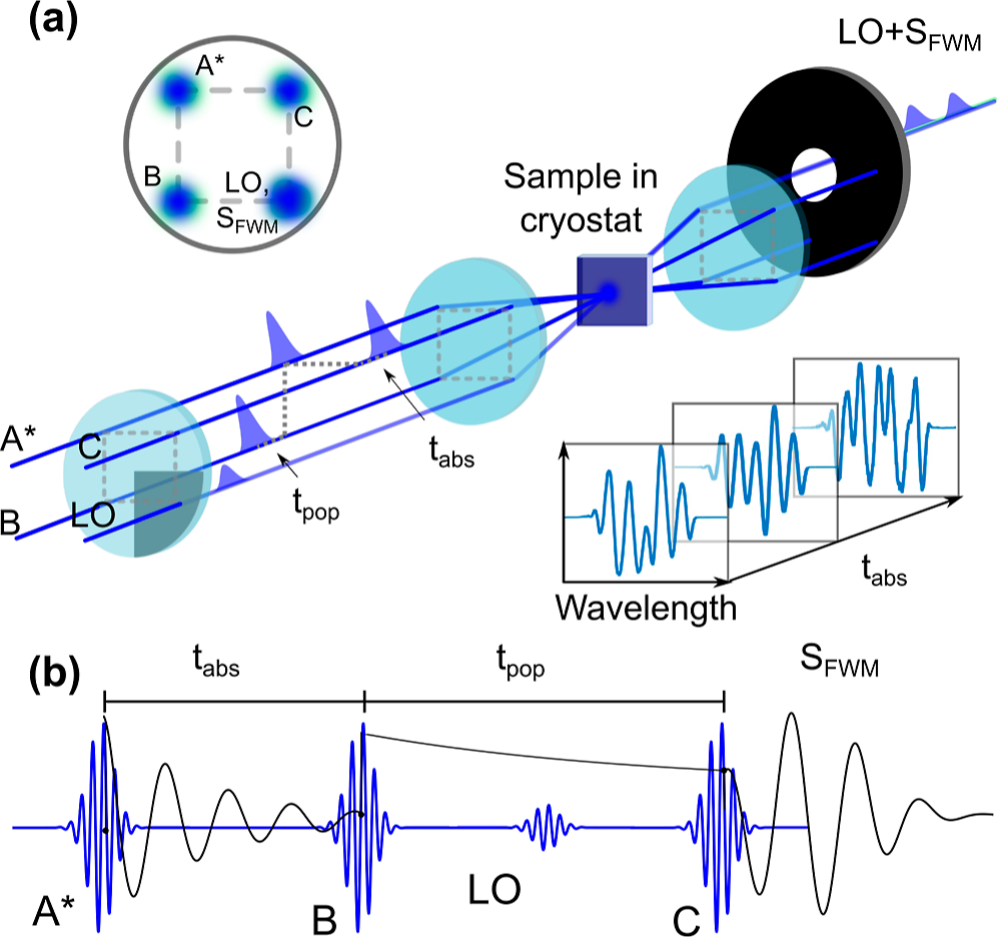
(a) Schematic representation of two-dimensional coherent electronic spectroscopy in a “boxcar” geometry. The experiment implements phase matching and time ordering of the three light–matter interactions, producing a third-order time-varying polarization that emits the coherent response, *S*_FWM_, along the fourth beam of the arrangement acting as a local oscillator (LO). *S*_FWM_ is detected by interference with a local oscillator. (b) Pulse train ordering corresponding to the rephasing experiment signal, with *k*-vector *k⃗*_FWM_ = −*k⃗*A + *k⃗*_B_ + *k⃗*_C_. Figure adapted with permission from ref (37). Published by the American Physical Society under the Creative Commons Attribution 4.0 International license.

In this paper, we present a study on the effect of metal cation substitution on the excitonic Coulomb interactions in hybrid RPMHs. We performed two-dimensional coherent electronic spectroscopy, Figure 1, on a prototypical tin-based RPMH, (PEA)_2_SnI_4_, to characterize the excitonic response. Specifically, we observed two distinct exciton signatures with different complex lineshapes and different EID behavior. To quantify the EID interaction parameter, we studied the linewidth dependence on excitation density. We also developed a microscopic interpretation of the phase-scrambled lineshapes using a stochastic scattering model and a biexciton excitation pathway, which helped us to understand the differences between the two exciton polaron states. We further compared our findings with our previous work on the lead counterpart, (PEA)_2_PbI_4_, and suggested that the differences in the nonlinear response could be attributed to their distinct degrees of static disorder. Overall, our results shed light on the effect of metal cation substitution on the excitonic response of RPMHs and provide insights into the underlying physics of these interesting materials.

## Results

### Exciton Linewidth Broadening

**Figure 2:**
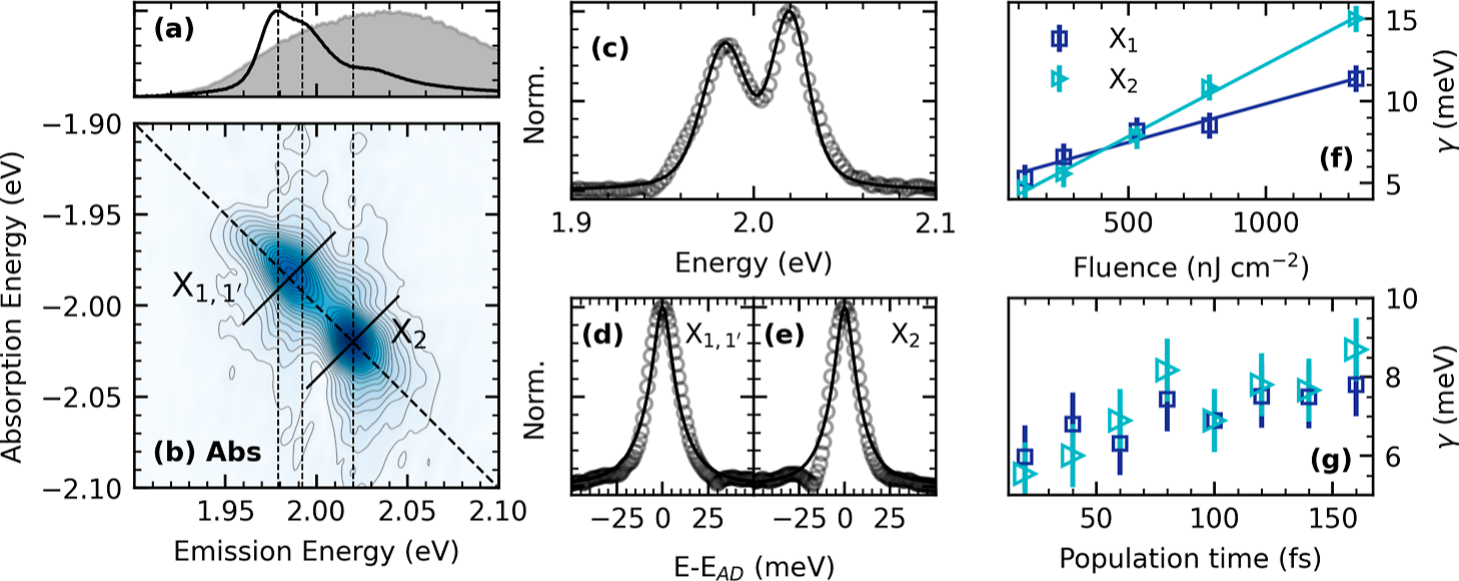
(a) Absorption spectrum of (PEA)_2_SnI_4_ (black line) measured at 15 K and pulse spectrum (gray filled curve). (b) Absolute rephasing 2D coherent spectrum measured at 15 K at a population waiting time of 20 fs. The dashed lines mark the approximate positions of the excitonic absorption at energies: 1.979, 1.992, and 2.020 eV. (c) Normalized diagonal cuts and antidiagonal cuts at the diagonal energies of excitons X_1,1′_ and X_2_, (d) and (e), respectively, where *E* – *E*_*AD*_ is the energy difference with the diagonal. (f) Fluence dependence and (g) population evolution of the homogeneous linewidth of *X*_1,1′_ and *X*_2_.

The (PEA)_2_SnI_4_ sample corresponds to a polycrystalline film prepared through spin coating. Further details on the preparation are described in the Supporting Information; additionally, the microstructure (SEM) and X-ray diffraction (XRD) characterizations are presented in the previous work.^10^ We first describe the main features observed in the linear and nonlinear absolute spectra. Three spectral features, labeled *X*_1_, *X*_1′_, and *X*_2_, can be clearly observed at the exciton energy in the linear absorption spectra, as shown in Figure 2a. In a two-dimensional Fourier transform experiment, a third-order polarization resonant with the exciton energy is generated by a sequence of three laser pulses incident on the sample in a “boxcar” geometry, as shown in Figure 1. The coherent emission due to this induced third-order polarization is then detected through spectral interferometry with a fourth attenuated pulse (local oscillator, LO) copropagated with the emitted field. Here, we focus on the analysis of the rephasing spectra where the emitted signal is acquired at *k⃗*_FWM_ = −*k⃗*_A_ + *k⃗*_*B*_ + *k⃗*_C_. In Figure 2b, we show the absolute value of the rephasing 2D coherent spectrum measured with a fluence of 178 nJ cm^–2^ per pulse. We observe two features along the diagonal corresponding to the energies of *X*_1_ and *X*_2_. Since *X*_1_ and *X*_1′_ are not well resolved, we refer to the corresponding feature in the 2D spectrum as *X*_1,1′_. We also observe an off-diagonal cross peak between *X*_1,1′_ and *X*_2_ with a very low intensity. Note that the cross-peak has a π phase-shift relative to the *X*_1,1′_ feature observed in the real and imaginary components of the total correlation spectra, Figure S4. This suggests that the feature is due to an excited-state absorption pathway from an exciton state to a higher energy state common to both excitons. A similar scenario was described for the case of (PEA)_2_PbI_4_^38^ where the higher state is assigned to a biexciton, which has been a subject of study for several years in the community.^39−41^ In the case of (PEA)_2_SnI_4_, we speculate that the excited-state absorption pathway involves a mixed biexciton between *X*_1,1′_ and *X*_2_. A detailed characterization of its binding energy and dephasing dynamics will be addressed in future work.

The elastic scattering events of excitons, e.g., exciton–exciton and exciton–phonon scattering, are characterized by their effect on the homogeneous linewidth, γ = Γ/2 + γ_0_ where Γ is the inverse of the exciton lifetime and γ_0_ is the pure dephasing term. The inhomogeneous nature of the semiconducting samples due to variations in the potential landscape leads to further broadening of the exciton excitation spectrum. From linear spectral measurement, we cannot rigorously separate the homogeneous and inhomogeneous contributions, we must therefore resort to nonlinear spectroscopy, particularly in materials such as RPMHs, which are highly dynamic.^42^ From an analysis of the diagonal and antidiagonal line widths of the rephasing 2D coherent spectra, we can extract the inhomogeneous and homogeneous broadening contributions.^43,44^Figure 2c shows a diagonal cut of the absolute rephasing map, while panels (d) and (e) show the antidiagonal cut. Since the homogeneous and inhomogeneous contributions are comparable and convoluted, the distinct contributions are extracted by simultaneously fitting the diagonal and antidiagonal to the expressions derived previously by Siemens and Bristow^14,44^ rather than simple Gaussian and Lorentzian functions. We obtained a homogeneous linewidth of 5.3 ± 0.8 and 4.7 ± 0.8 meV for *X*_1,1′_ and *X*_2_, respectively. The linear fluence dependence of the linewidth shown in Figure 2f, clearly shows that excitons *X*_1,1′_ and *X*_2_ are both subject to EID due to interexciton interactions. The strength of the interaction is quantified by the slope of the fluence dependence fitted to eq 3,^43,45^ where Δ_ex_ is the interaction parameter and *n* is the excitation density
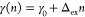
3

We obtain a Δ_ex_ of 2.4 × 10^–8^ and 4.5 × 10^–8^ μeV cm^2^ for exciton *X*_1,1′_ and *X*_2_, respectively. We highlight that these values are orders of magnitude larger than the interaction parameters obtained previously for (PEA)_2_PbI_4_.^37^ The observed difference in the exciton–exciton interaction parameters between (PEA)_2_PbI_4_ and (PEA)_2_SnI_4_ is striking. We have suggested previously that many-body interactions are possibly screened by the lattice dressing of the excitations. Such polaronic effects are not expected to change drastically, moving from lead to tin, enough to quench the screening over 3 orders of magnitude. We hypothesize that the increased interactions in the (PEA)_2_SnI_4_ are instead a consequence of the increased exciton dipole moment, quantified recently by Hansen et al.^34^ by electroabsorption spectroscopy. They reported that the dipole moment in the tin system is seven times larger in comparison to the lead counterpart and attributed it to reduced dynamic disorder within the lattice. In the same context, efficient dielectric screening has been linked to a dynamic distortion due to the stereochemical expression of *ns*^2^ electron pairs,^36,46^ when substituting Pb^2+^ with Sn^2+^, the distortion becomes less dynamic (more static) and might result in the stabilization of charge separated excitons.^46^ The difference in the EID between lead and tin might arise from differences in the dynamic nature of the structure.

Another distinction between the lead and tin systems can be observed in the evolution of the homogeneous linewidth (γ) with population waiting time (delay between pulses B and C). In the case of (PEA)_2_PbI_4_, we observed a rather peculiar reduction in the linewidth with time, which we interpreted as a nonstationary evolution of the interaction with the dark background population. Here, contrary to our earlier observation, we see that the linewidth increases, albeit moderately, with population waiting time for (PEA)_2_SnI_4_, as shown in Figure 2g. Such line broadening is typically attributed to spectral diffusion^47,48^ with the photoexcited coherent population accessing the inhomogeneous energy distribution that stems from static lattice disorder. This is also quantified by the diagonal linewidth of δω = 10 ± 1 meV from the fits in Figure 2c, which is much larger than the estimates for (PEA)_2_PbI_4_ (δω = 6.6 ± 0.1 meV). A larger static disorder, which may vary significantly from sample to sample and might also be related to Sn^2+^ oxidation to Sn^4+^,^49^ does not allow us to visualize the time evolution in the screening of interexcitonic interactions, which cannot be discounted in (PEA)_2_SnI_4_.

### Temperature-Dependent Broadening

**Figure 3:**
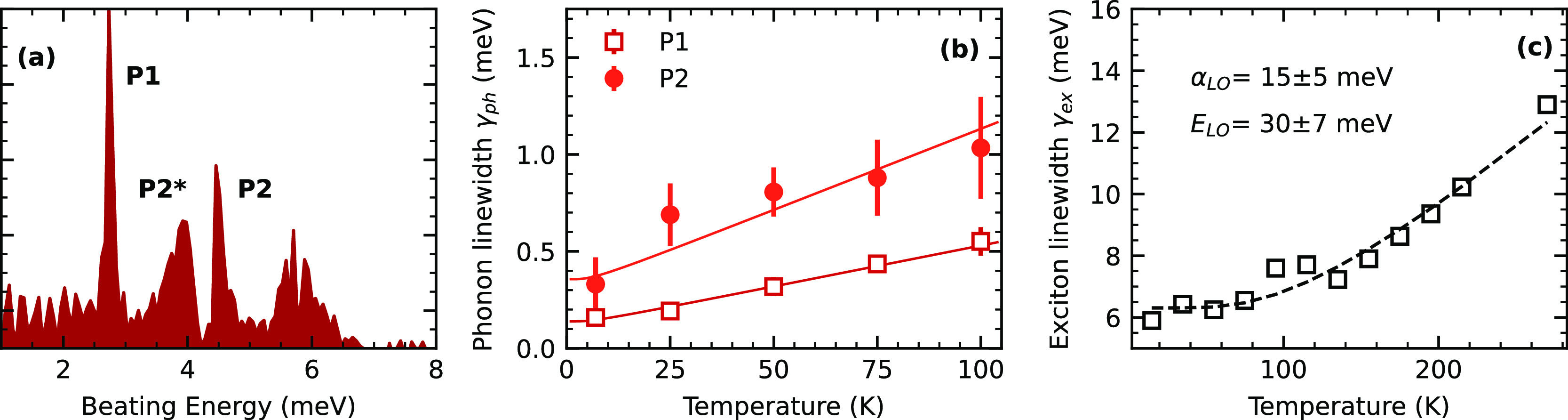
For (PEA)_2_SnI_4_. (a) Cumulative RISRS spectrum measured at 7 K. (b) Temperature dependence of the dephasing time for the modes *P*_1_ and *P*_2_, the solid line is fit to the cubic overtone equation. (c) Temperature dependence of the X_1,1′_ homogeneous linewidth, obtained from the temperature-dependent linewidth analyzed by 2D coherent electronic spectroscopy.

As mentioned above, we observed an enhanced exciton–exciton interaction parameter for the case of (PEA)_2_SnI_4_. We then are urged to study the anharmonicity and exciton–phonon coupling, aiming to pinpoint the enhanced Δ_ex_ to differences in the lattice-carrier coupling. Resonance impulsive stimulated Raman scattering (RISRS) is an effective alternative to a cw resonance Raman, particularly for estimating the low energy phonon modes that dress photoexcitations in highly emissive materials with small Stokes shifts. The evolution of a coherent superposition of phonon modes, generated through an impulsive excitation with an ultrashort optical pulse, modulates the complex refractive index. We observe such a modulation as a time-oscillating differential transmission signal with characteristic lineshapes. An exhaustive description of the physical processes responsible for the generation of the coherent wave packet and distinct experimental detection approaches can be found in refs (50) and (51), and for the case of RPMHs in ref (18). In our previous work, we determined that the RISRS spectrum of (PEA)_2_PbI_4_ is primarily composed of two phonon modes with energies 2.61 and 4.40 meV.^17^ They were assigned to a pseudocubic axis, octahedral twist, and Pb–I–Pb bending, respectively. In nonresonant impulsive stimulated Raman scattering, similar modes have been observed at 3 and 6 meV in (BA)_2_PbI_4_ and 3 meV in MAPbI_3_, then later assigned to antiphase octahedra rotations.^52^ We obtained the cumulative resonance Raman spectra of a (PEA)_2_SnI_4_ thin film from the modulation of the ground state bleach by Fourier transforming and binning the respective time traces, Figure 3a. We show the time traces and Fourier transform maps in the Supporting Information. We observe, unsurprisingly, a similar spectrum with an additional Raman mode below 4 meV, labeled as *P*_2_*, as also reported recently in ref (32). We hypothesize that *P*_2_* arises due to the static distortion of (PEA)_2_SnI_4_ although it is hard to assign the origin of the static disorder due to the presence of self-doped domains in the thin film due to progressive oxidation of the Sn^2+^ to Sn^4+^.

The temperature dependence of the phonon linewidth is directly related to anharmonic effects (phonon–phonon interaction), which becomes more significant as the phonon populations increase, as shown in Figure 3b. We interpret the phonon–phonon interactions in terms of the overtone decay mechanism described by 4(53,54)
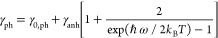
4

γ_0,ph_ corresponds to a temperature-independent rate which includes defect scattering^55^ and γ_anh_ is the anharmonic coefficient. We obtain for *P*_1_ a γ_anh_ = 0.016 ± 0.1 ps^–1^ and *P*_2_ a γ_anh_ = 0.05 ± 0.01 ps^–1^. In order to compare across modes and materials, we obtain the dimensionless parameter ω/2πγ_0_, shown in Table 1. As observed for the case of (PEA)_2_PbI_4_,^56^*P*_1_ is less anharmonic than *P*_2_, and in general, we note that the modes observed in tin are slightly more anharmonic than the lead counterpart. Independently, the temperature dependence of the exciton linewidth broadening provides the means of quantifying exciton–phonon coupling typically described by eq 5
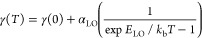
5

**Table 1:** Summary of Parameters Extracted in This Work for (PEA)_2_SnI_4_ and Previously Obtained for (PEA)_2_PbI_4_ from Refs (37) and (56)^a^

Parameters	Value for **(PEA)**_**2**_**SnI**_**4**_	Value for **(PEA)**_**2**_**PbI**_**4**_
ω_ph_/2πγ_0_ (P_1_/M_1_)	42 ± 5	
ω_ph_/2πγ_0_ (P_2_/M_2_)	21 ± 9	35 ± 6
Δ_ph_ (μeV/K)	44 ± 20	70 ± 20
*E*_LO_ (meV)	30 ± 7	6.5 ± 0.9

a We use the labels M_1_ and M_2_ from previous work to refer to the phonon modes in (PEA)_2_PbI_4_. The parameters recovered for the exciton-phonon coupling for (PEA)_2_PbI_4_ correspond to the exciton label as A in ref (37), the exciton with energy (2.37 eV).

In Figure 3c, we show the dependence of the X_1,1′_ dephasing with temperature determined through coherent spectroscopy. The intensity of X_2_ decreases as temperature increases and cannot be resolved unambiguously; therefore, the temperature dephasing is not shown in this work. We estimate the interaction parameter, Δ_ph_, as Δ_ph_ = α_LO_*k*_B_/*E*_LO_. Its value is comparable to that determined for (PEA)_2_PbI_4_, summarized in Table 1. We note that in previous work, temperature-dependent X-ray diffraction experiments indicated that there is not a phase transition for either the (PEA)_2_PbI_4_ or (PEA)_2_SnI_4_ film materials.^10,38^

We note that eq 5 has been widely used for temperature-dependent linewidth analysis from linear photoluminescence measurements. However, it presents several limitations; specifically, the model considers coupling with a single harmonic mode and does not consider short-range lattice coupling interactions. This is contrary to the strong anharmonicity reported in the Ruddlesden–Popper phases.^56,57^ Additionally, in a previous perspective,^42^ we argued that due to the inhomogeneous nature of RPMHs, the steady-state photoluminescence lineshape is not strictly temperature-independent if driven by diffusion-limited processes. The temperature-dependent population of dark states^22,35,58,59^ might also play a role in the temperature-dependent dephasing of the excitonic features. For all these reasons, we restrain ourselves from attributing a robust physical meaning to the parameters extracted and from comparing with the many examples in the literature.^22,34,60^

In this section, we characterized the exciton–phonon coupling and the lattice anharmonicity. The results are not surprising, describing a very similar scenario for (PEA)_2_SnI_4_ compared to its lead counterpart, with just minor variations in the interaction parameters determined. This analysis discards a significant difference in the polaronic screening as the source of the very distinct exciton–exciton interaction parameter. The enhanced exciton–exciton interaction parameter could be related to an extrinsic factor, for example, as described above due to the enhanced strength of the exciton dipole moment induced by a disordered lattice,^34^ or exciton-carrier scattering due to the unintentional doping of tin perovskites.^61^

### Complex Lineshape Analysis and Stochastic Scattering Modeling

The linewidth analysis of the absolute value of the rephasing spectrum, presented in the sections above, presents two important observations regarding the many-body interactions in (PEA)_2_SnI_4_: (i) the interactions between excitons are much enhanced, possibly due to enhanced strength of the exciton dipole moment, and (ii) the sample under study exhibits larger static lattice disorder. The first observation may be a direct consequence of the metal cation substitution, while the second one may also be a result of the variability in the sample fabrication conditions. While holding on to these observations, we now turn our attention to the details in the spectral lineshape of real and imaginary components of the rephasing spectrum. As noted in the Introduction of this paper, the relative phase shifts in the nonlinear response, which manifest as deviations from expected absorptive and dispersive lineshapes in the real and imaginary components, respectively, provide deeper insights into the origins of the many-body scattering.^24,28,62^

**Figure 4:**
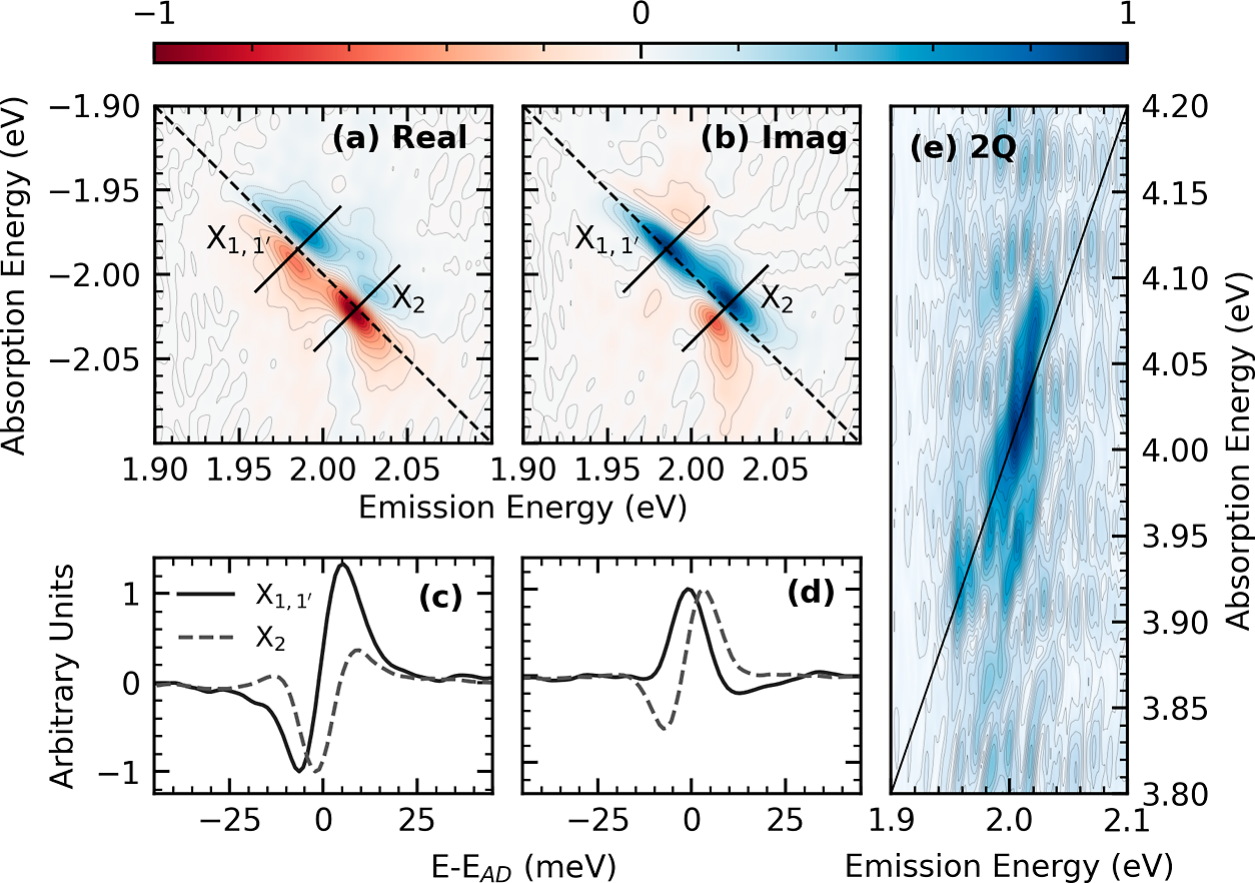
(a) Real and (b) imaginary components of the rephasing 2D coherent spectra measured at 15 K at a population waiting time of 20 fs, phased at 1.983 eV. Antidiagonal cuts of the (c) real and (d) imaginary components showing the two main excitonic components *X*_1,1′_ (solid line, black) and *X*_2_ (dashed line, gray). (e) Two-quantum nonrephasing 2D coherent excitation absolute measurement of (PEA)_2_SnI_4_.

The real and imaginary components of the rephasing 2D spectra are shown in Figure 4a,b, respectively. We observe that the real part of the spectrum is clearly dispersive at the energy of exciton *X*_1,1′_, with a positive slope along the antidiagonal line and zero signal along the diagonal. The associated imaginary component is absorptive. The lineshape at *X*_1,1′_ is similar to what we have previously reported for excitons in (PEA)_2_PbI_4_, and much like in that case, we interpret it as a signature of EID due to interaction with the background excitations. This is consistent with the fluence dependence of the homogeneous linewidth (see Figure 2f) that resulted in a larger exciton–exciton scattering parameter. The lineshape at *X*_2_ in Figure 4, however, does not follow this behavior. The real part at *X*_2_ looks absorptive but with a bit of asymmetry that shifts the positive peak to lower energies along the antidiagonal line. The imaginary component looks dispersive, as expected for an isolated noninteracting system. This is absolutely not consistent with the estimated interaction parameter from the linewidth analysis for *X*_2_. We consider that this discrepancy is not due to the failure of our photophysical model but due to the overlapping contribution from the excitation pathway to a biexcitonic state, which offsets the phase shift in the signal induced by many-body interactions. This will be further discussed in the next section based on simulations of the nonlinear response using stochastic scattering theory.

Before dwelling into the simulations, we first independently verify the presence of a biexcitonic state associated with *X*_2_ using a coherent two-quantum (2Q) measurement.^63,64^ The emitted field is collected at *k⃗*_FWM_ = *k⃗*_B_ + *k⃗*_C_ – *k⃗*_A_, where the conjugated pulse interacts last. The outcome is a 2D map correlating with the energy of one-quantum and two-quantum excitations. The presence of the biexciton state can be confirmed by the appearance of a feature below the diagonal line in the 2D map, as shown in Figure 4e. We clearly observe such a feature below 10 meV at the energy of *X*_2_ indicating a biexciton with a very low binding energy. Notably, no such feature is observed at the energy of *X*_1,1′_. The binding energy is much lower in (PEA)PbI_4_,^38^ which will result in a substantial overlap of excitation pathways of exciton and its biexciton.

We modeled the nonlinear response using the stochastic exciton scattering model developed in refs (28),^65^,^66^. The model accounts for the nonstationary evolution of the population of dark background excitations, characterized by the initial average population density *N*_0_ and variance σ_*N*_^2^. The nonstationary population is generated by each broadband excitation pulse and coupled to the optical mode via Coulombic interactions and evolves according to a stochastic differential equation corresponding to an Ornstein–Uhlenbeck process with damping rate Γ and variance σ^2^. The parameters in the model are related to the exciton–exciton interactions and, in principle, can be determined by ab initio or density functional theory methods. Here, we treat the exciton–exciton interaction *V*_0_ as adjustable parameters to examine how changes in the many-body interaction strength manifest in the nonlinear optical response. The most important components of the model are summarized in the Supporting Information. We consider independent transitions insofar as our theoretical model is concerned.

**Figure 5:**
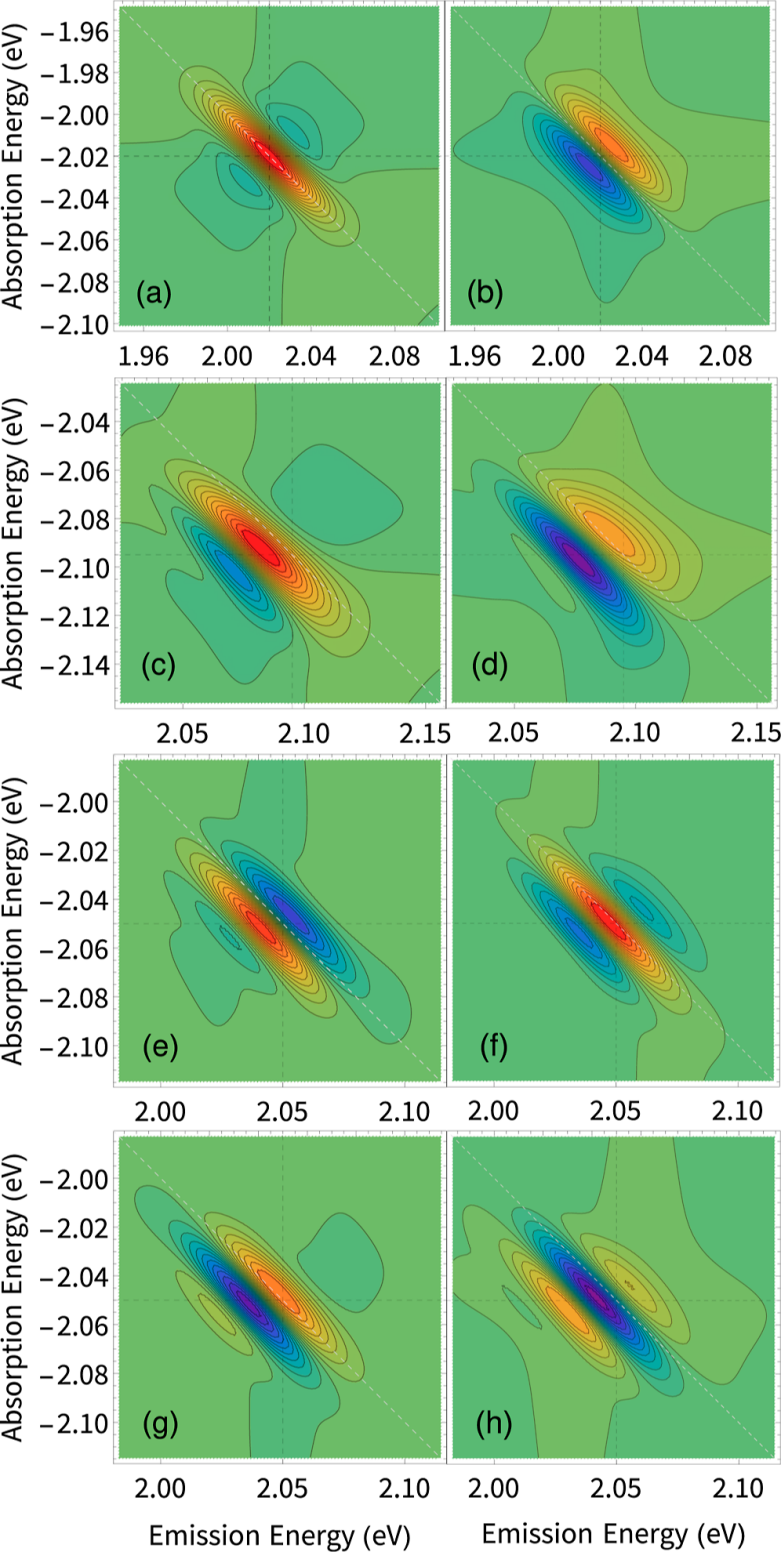
(a) Real and (b) imaginary components of a calculated rephasing spectrum with a background excitation density (*N*_0_ = 0). (c) Real and (d) imaginary components with a background excitation density (*N*_0_ = 0.2), without the biexciton contribution. (e) Real and (f) imaginary components with background excitation density (*N*_0_ = 0.2), with the biexciton contribution with *V*_0_ = 5 mV. (g) Real and (h) imaginary components with background excitation density (*N*_0_ = 0.2), with the biexciton contribution with *V*_0_ = −5 mV. The background is modulated by an Ornstein–Uhlenbeck process of damping rate γ = 1.2 ps^–1^ ≈ 5 meV and fluctuation variance σ^2^ = 25 ns^–1^.

**Figure 6:**
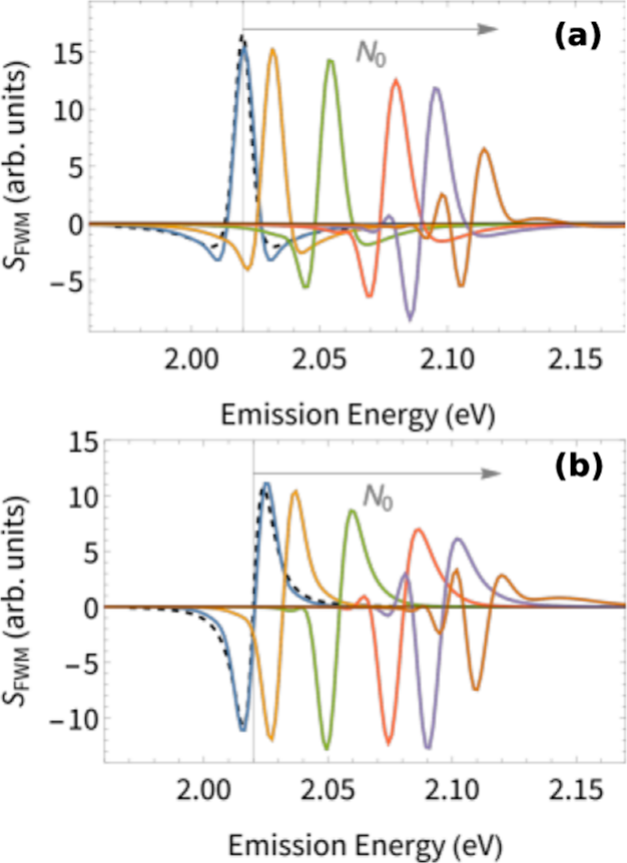
Effect of background exciton density *N*_0_ on the homogeneous lineshape of the coherent four-wave mixing signal *S*_FWM_. The antidiagonal cut is taken at the strongest spectral peaks *N*_0_ = 0, 0.1, 0.3, 0.5, 0.7, and 1.0 from left to right. The black dashed curve is fitted from eq 2, while the vertical grid line marks the energy of the bare exciton. The real component of the 2D coherent spectra and (b) imaginary component.

When the initial population is set to zero (*N*_0_ = 0), the model produces the rephasing spectra shown in Figure 5a,b, with symmetric lineshapes as expected for an isolated transition (cf. Equation 1). Introducing a finite background population with *N*_0_ = 0.2 that produces the asymmetric line shapes shown in Figure 5c,d, reproducing qualitatively the experimental observation for *X*_1,1′_. The asymmetry results from the additional phase factor that arises from the EID processes. This is evidenced in Figure 6a,b, where we show the antidiagonal cuts of the simulated rephasing spectra. We observe a clear evolution of the antidiagonal of the simulated spectrum as we increase the interacting background population. Note that the real component transitions from an absorptive-like lineshape when there is not an interacting background to a dispersive-like lineshape as the *N*_0_ increases. The imaginary component has the opposite behavior, resulting in an apparent π/2 phase shift in the spectrum. We note that the model can inherently account for the effect of exciton self-interaction (that leads to the biexciton) as a cross-peak whose position relative to the exciton diagonal feature is determined by the self-interaction amplitude *V*_0_. This contribution is, however, not considered for the simulations shown in Figure 5c,d, given the lack of a clear biexciton signature for the exciton *X*_1,1′_ in the two-quantum spectrum in Figure 4e.

To model the experimental lineshape corresponding to *X*_2_, we incorporate the biexciton pathways by setting the exciton self-interaction to *V*_0_ = ± 5 meV. Note that a positive *V*_0_ indicates a repulsive biexciton interaction and a negative *V*_0_ indicates an attractive interaction. Above, we mentioned that the biexciton pathway results in an emitted electric field with a phase factor of π compared to that of the exciton pathway. For a low biexciton binding energy, the diagonal exciton feature overlaps with the off-diagonal biexciton cross-peak, producing a complicated lineshape. The overlap of two dispersive-like lineshapes with a phase difference of π results in an apparent absorptive-like lineshape. This is shown in Figure 5e,f, with *V*_0_ = 5 meV, and Figure 5g,h, with *V*_0_ = −5 meV. Observe that including the biexcitonic term imparts an apparent phase shift of ± π/2 depending on the biexciton’s binding energy. We refer to the phase shift as apparent since it is due to the overlap of two closely spaced spectral features, and thus, the overall phase shift is not a real physical consequence. To summarize, the spectrum shown in Figure 5c,g qualitatively reproduced the experimental spectrum of *X*_1,1′_ and *X*_2_, respectively, as shown in Figure 4a.

## Discussion

As stated in the introduction, we conducted a study of the coherent nonlinear response of (PEA)_2_SnI_4_, a tin-based RPMH, to comprehend the crucial role of the ionic lattice in many-body Coulomb interactions. Our investigation led to three significant differences in (PEA)_2_SnI_4_ compared to (PEA)_2_PbI_4_: (i) a notably larger exciton–exciton interaction parameter Δ_ex_,^37^ (ii) higher static disorder,^16^ and (iii) lower biexciton binding^38^ energy, albeit with a higher transition cross-section. Moreover, the anharmonicity of the lattice and exciton–phonon coupling of (PEA)_2_SnI_4_ are both comparable with those determined for (PEA)_2_PbI_4_ discarding a lack of polaronic screening as the origin of the observed differences.

The enhanced exciton–exciton interactions are discernible in the fluence-dependent exciton dephasing rates and the characteristic spectral lineshapes of the real and imaginary components of the rephasing spectra. We hypothesize that it can be attributed to the increased exciton dipole moment. Interestingly, some researchers have suggested the possibility of a disorder-induced transition dipole moment enhancement,^67^ based on a comparison of the transition dipole moments of tin- and lead-based RPMH.^34^ We observe a more static-disordered structure for the case of (PEA)_2_SnI_4_, which can be observed in the population time evolution of the nonlinear spectra and the broad additional phonon mode in the Raman spectra. The description of the static disorder is significant as it impacts the excitonic properties of the material by inducing localized excitons through backscattering of the wave packet from defect sites.^47^

Exciton-carrier scattering^68−70^ is an additional scattering pathway that has not been addressed in this work explicitly. It possesses fundamentally a higher interaction strength than exciton–exciton interactions, as it can be understood as a monopole interacting with a dipole instead of dipole–dipole interaction. For example, early work in GaAs single quantum wells determined that exciton–carrier scattering is 8 times stronger than exciton–exciton interactions.^71^ In the experiments presented here, we do not explore this interaction, as we do not pump the free carriers with our pulse spectrum. Any carrier population from unintentional doping is not expected to result in the observed fluence dependence. Accordingly, we discount the contribution of exciton-carrier scattering to EID. An interesting avenue to study this interaction may be available as the materials community further explores the doping^61,72^ of RPMHs.

We anticipate that the increased exciton–exciton interactions would also lead to an increase in the binding energy of the biexcitons, as the strength of the Coulomb coupling fundamentally governs both processes. However, contrary to such an expectation, we observe that the biexciton binding energy in (PEA)_2_SnI_4_ is below 10 meV, while it is about 50 meV in (PEA)_2_PbI_4_. This highlights the mechanistic differences between the elastic many-body scattering of excitons and biexciton binding. The former can be interpreted as dipole–dipole scattering with the interaction strength proportional to the physical dipole’s strength. In contrast, the biexciton results from four-particle (two electrons and two holes) correlations, and its binding energy is determined by the relative attraction and repulsion between charge carriers in the lattice. We emphasize that the biexciton feature can be observed in the rephasing spectrum of (PEA)_2_SnI_4_ at relatively low excitation density, while we required 2 orders of magnitude higher excitation density to observe it in (PEA)_2_PbI_4_.^38^ This can be attributed to the larger exciton-to-biexciton transition cross-section. However, it should be noted that in addition to lattice coupling, other physical phenomena, such as exchange interactions, may also influence the strengths of exciton–exciton interactions. Spin-selective lineshapes on excited state absorption features assigned to repulsive and attractive biexciton interactions have been observed in differential transmission spectroscopy following the exciton spin dynamics of lead-bromide perovskite nanoplatelets^73^ and (PEA)_2_PbI_4_.^74^ However, we have not explored this framework in our current work. Multidimensional spectroscopic experiments with circularly polarized light would significantly enhance our understanding of biexcitonic interactions.^63^ In our study, we demonstrated how phase shifts can occur as a result of interactions of exciton with the background population. However, overlapping features, in this case, due to a biexcitonic excitation pathway, can also cause apparent phase shifts that are difficult to interpret without additional experiments, such as two-quantum excitation pathways.

## Conclusions

We employed two-dimensional Fourier transform spectroscopy to investigate the excitons in (PEA)_2_SnI_4_. By performing a comprehensive analysis of linewidth and the complex line shape, we identified differences in the many-body exciton interactions between the tin and lead samples. These differences provide insights into important chemical variables that could be manipulated to tune the exciton nonlinear interactions. We propose that subtle structural modifications in RPMHs, where the lattice plays a deterministic role in the excitonic properties, could provide experimental access to distinct degrees of the exciton–exciton interaction. This could be achieved by exploring the parameter space involving static and dynamic structure, dimensionality, chemical composition, and spin–orbit coupling strength. In this work, we observed that metal substitution resulted in an enhanced exciton interaction compared to its lead counterpart, and we observed an additional biexcitonic feature with low binding energy (<10 meV).

## Supporting Information Available

Experimental procedures, additional characterization, and details regarding the model to simulate the nonlinear response (PDF)
