# Supplementary material for: Many-Exciton Quantum Dynamics in a Ruddlesden–Popper Tin Iodide
Source: J Phys Chem C Nanomater Interfaces. 2023 Oct 25;127(43):21194–203. doi: 10.1021/acs.jpcc.3c04896 (PMC10626601; doi:10.1021/acs.jpcc.3c04896)
Supplement: Supplementary file 1 — jp3c04896_si_001.pdf [file jp3c04896_si_001.pdf]

Many-Exciton Quantum Dynamics in a Ruddlesden-Popper Tin Iodide

Esteban Rojas-Gatjens[1,2], Hao Li[3], Alejandro Vega-Flick[1], Daniele Cortecchia[4,5], Annamaria Petrozza[4], Eric R. Bittner[3,6], Ajay Ram Srimath Kandada[7], Carlos Silva-Acuña[‡1,2,8]

[1] *School of Chemistry and Biochemistry, Georgia Institute of Technology, Atlanta, GA, United States.*

[2] *School of Physics, Georgia Institute of Technology, Atlanta, GA, United States.*

[3] *Department of Chemistry, University of Houston, Houston, Texas 77204, United States.*

[4] *Center for Nano Science and Technology@PoliMi, Istituto Italiano di Tecnologia, Italy.*

[5] *Dipartimento di Chimica Industriale Toso Montanari, Università di Bologna, 40136 Bologna, Italy.*

[6] *Center for Nonlinear Studies, Los Alamos National Laboratory, United States.*

[7] *Department of Physics and Center for Functional Materials, Wake Forest University, Winston-Salem, NC, United States.*

[8] *School of Materials Science and Engineering, Georgia Institute of Technology, Atlanta, GA, United States*

Contents

1 Experimental Methods **S2**
- 1.1 Sample preparation . . . S2
- 1.2 Two-dimensional coherent electronic spectroscopy . . . S2
- 1.3 Ultrafast Transient Absorption Spectroscopy . . . S2

2 Ultrafast differential Transmission **S2**
- 2.1 Resonant Impulsive Raman Scattering . . . S2
- 2.2 Wavelet Analysis Spectrograms . . . S4

3 Multidimensional spectroscopy **S5**
- 3.1 Total correlation two-dimensional spectrum . . . S5
- 3.2 Temperature dependence 2D spectra . . . S6
  - 3.2.1 Population dependence 2D spectra . . . S7
- 3.3 Linewidth analysis . . . S7
- 3.4 Fitting procedure . . . S7
- 3.5 Pulse duration characterization . . . S7

4 Stochastic scattering model **S9**

1 Experimental Methods

1.1 Sample preparation

The samples were prepared by mixing the precursors phenylethylammonium iodide (PEAI, Gratcell Solar Materials) and SnI_2 (TCI) in 2:1 stoichiometric ratio to form 0.05 M solutions in dimethylformamide (DMF, Anhydrous, Sigma Aldrich). The powders were left to dissolve overnight, then the solution was heated up to 100 °C for 1 hour before the deposition process. $(PEA)_2SnI_4$ polycrystalline thin films were spin-coated from the hot solution at 5000 rpm on fused silica substrates. The film was then annealed at 100 °C for 15 minutes on a hotplate. The entire process was performed under an inert atmosphere in a N_2 filled glove box. The films were encapsulated using a glass slide and a UV-cured resin. The resin was deposited only on the sample's edges to prevent direct contact with the central part of the sample where the measurements were performed. The perovskite film was masked during the UV-curing process to prevent its direct exposure to UV light.

1.2 Two-dimensional coherent electronic spectroscopy

In this work, we used our previously implemented scheme [1], [2], developed and described in detail by Turner and coworkers [3], to measure the 2D coherent spectra. The 1030-nm, 220-fs pulse train was generated in a commercial ultrafast laser system (Light Conversion Pharos) at a 100-kHz repetition rate. A portion of the laser beam was sent into a home-built third-harmonic-pumped non-collinear optical parametric amplifier. The pulses were individually compressed using a home-built implementation of a pulse shaper using a chirp scan [4]. The resulting pulse duration was 21 fs full-width at half-maximum, as measured by second-harmonic generation cross-frequency-resolved optical gating (SHG-XFROG). All measurements were carried out in a vibration-free closed-cycle cryostat (Montana Instruments).

1.3 Ultrafast Transient Absorption Spectroscopy

Using an ultrafast laser system (Pharos Model PH1-20-0200-02-10, Light Conversion) emitting 1030 nm pulses at 100 kHz, with an output power of 20 W and pulse duration of 220 fs. The measurements were carried out in a commercial set-up (Light Conversion Hera). The pump wavelength 3.06 eV was generated by feeding 10 W from the laser output to a commercial optical parametric amplifier (Orpheus, Light Conversion), while 2 W are focused onto a sapphire crystal to obtain a single-filament white-light continuum covering the spectral range 490–1050 nm for the probe beam. All measurements were carried out in a vibration-free closed-cycle cryostat (Montana Instruments).

2 Ultrafast differential Transmission

2.1 Resonant Impulsive Raman Scattering

The electronic dynamics are subtracted from the differential transmission spectra using a polynomial fit. The resulting modulated response is then fast Fourier transformed at each probe-energy. The cumulative RIRS spectrum shown in the main text is obtained by binning the beating maps. The maps are shown in figure S2.

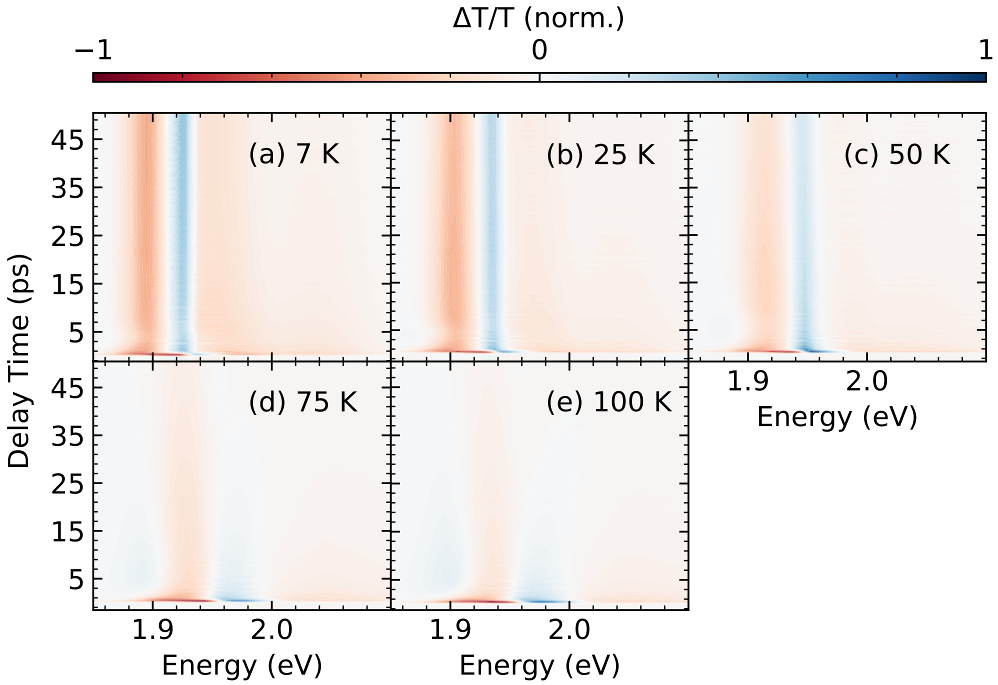

Figure S1: Time-resolved differential transmission spectra for $(PEA)_2SnI_4$ at temperatures (a) 7 K, (b) 25 K, (c) 50 , (d) 75 K and (e) 100 K.

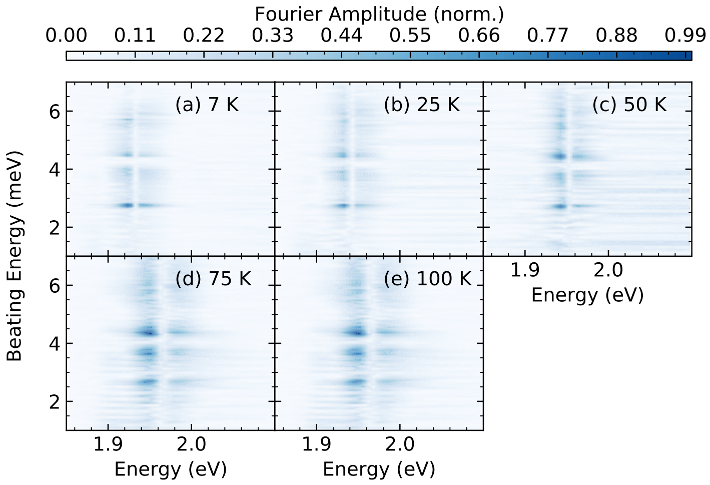

Figure S2: Resonant Impulsive Raman spectrum for $(PEA)_2SnI_4$ at temperatures (a) 7 K, (b) 25 K, (c) 50 , (d) 75 K and (e) 100 K.

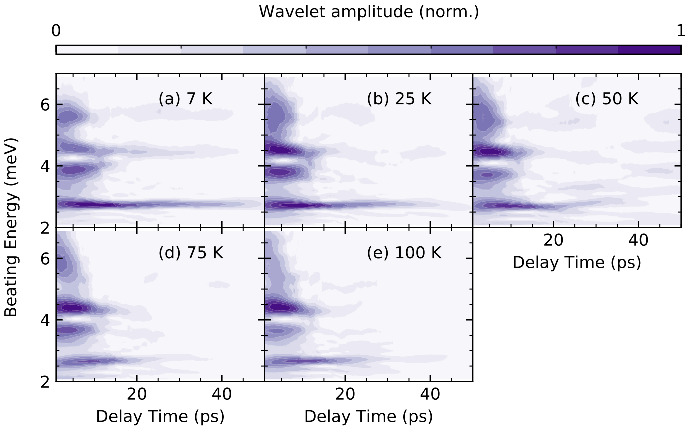

Figure S3: Wavelet transformation (CWT) spectrum of the binned time-domain RIRS data with a complex Morlet wavelet for $(PEA)_2SnI_4$ at several temperatures.

2.2 Wavelet Analysis Spectrograms

The CWT was implemented using the open-source wavelet transform software for Python under an MIT license. A complex Morlet wavelet was employed for the analysis, equation S1, with parameters B and C equal to 50 and 1 respectively. The wavelet transform is defined as equation S2, where $W(\tau, s)$ are the wavelet coefficients. The maps are shown in figure S3.

$$\psi\left(t\right) = \exp\left(-\frac{t^2}{B}\right)\exp\left(i2\pi Ct\right) \tag{S1}$$

$$W(\tau, s) = \frac{1}{|s|^{1/2}}\int_{-\infty}^{\infty} f\left(t\right)\ \psi^*\left(\frac{t-\tau}{s}\right)\ dt \tag{S2}$$

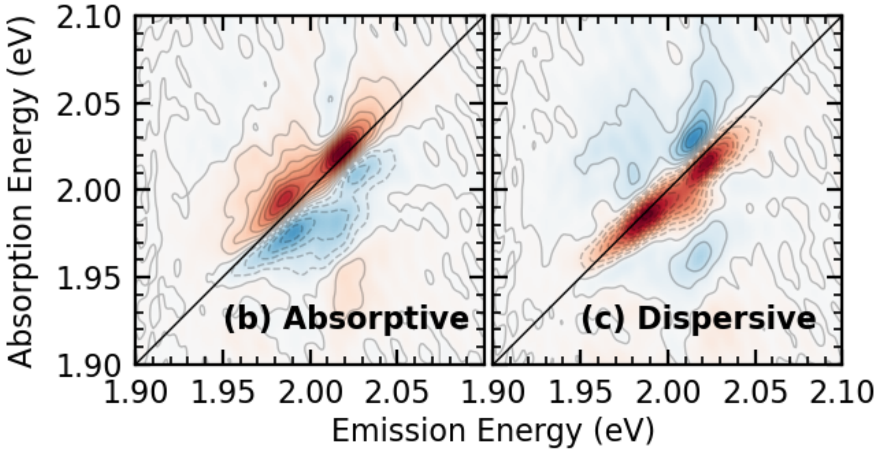

Figure S4: Total Correlation spectra (a) Absorptive and (b) dispersive components of the 2D spectra measured at 15 K at a population time of 20 fs, and phased at 1.983 eV.

3 Multidimensional spectroscopy

3.1 Total correlation two-dimensional spectrum

The total correlation spectra are recovered as:

$$Abs = \Re(Rep) + \Re(Nonrep), \tag{S3}$$

$$Dis = \Im(Rep) + \Im(Nonrep). \tag{S4}$$

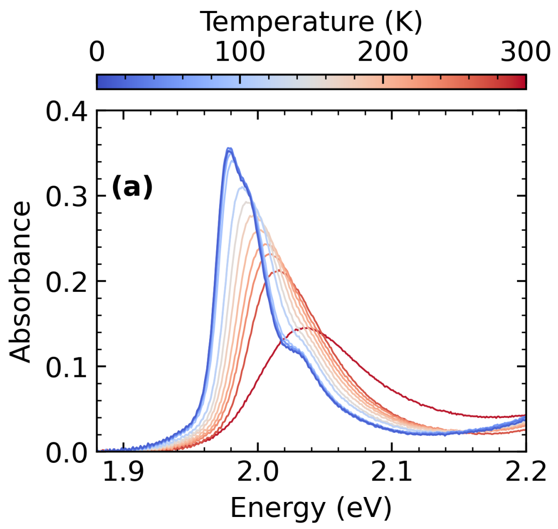

Figure S5: (a) Temperature-dependent absorption spectra for $(PEA)_2SnI_4$ films.

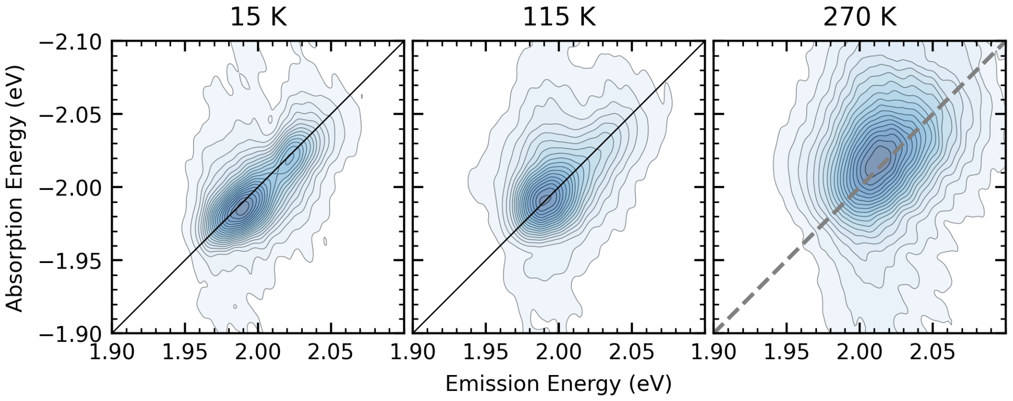

Figure S6: Absolute component of the rephasing 2D spectra at three distinct temperatures.

3.2 Temperature dependence 2D spectra

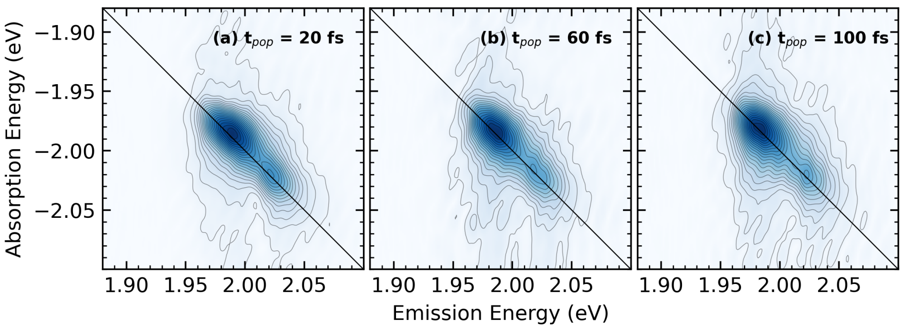

Figure S7: Absolute rephasing spectra at populations times (a) 20 fs, (b) 60 fs, and (c) 80 fs.

3.2.1 Population dependence 2D spectra

3.3 Linewidth analysis

Following the work by Siemens [5] and previous work [2]. We used the following expressions for extracting the homogeneous and inhomogeneous linewidths from the 1Q rephasing experiments.

$$S_{AD}(\omega_{ad}) = \left| \frac{\exp\left(\frac{(\gamma - i\omega_{ad})^2}{2\delta\omega}\right) \operatorname{erfc}\left(\frac{\gamma - i\omega_{ad}}{\sqrt{2}\delta\omega}\right)}{\delta\omega(\gamma - i\omega_{ad})} \right| \tag{S5}$$

$$\begin{aligned} S_D(\omega_d) = \sum_j \alpha_j \Bigg| & \frac{\exp\left(\frac{[\gamma - i(\omega_d - \omega_j)]^2}{2\delta\omega^2}\right)}{\gamma\delta\omega} \left[\operatorname{erfc}\left(\frac{\gamma - i(\omega - \omega_j)}{\sqrt{2}\delta\omega}\right)\right] \\ & + \exp\left(\frac{2\gamma i(\omega_d - \omega_j)}{\delta\omega^2}\right) \operatorname{erfc}\left(\frac{\gamma + i(\omega_d - \omega_j}{2\delta\omega}\right) \Bigg| \end{aligned} \tag{S6}$$

3.4 Fitting procedure

As noted from both the temperature dependence and fluence dependence the relative amplitude of X_2 makes the global fit unreliable as the inhomogeneous and homogeneous linewidth become correlated. We do global fit for measurements at two spots in the sample and obtain similar results, the inhomogeneous broadening parameter as well as the intensity changed in the new position. The global fit results are shown in table S1

3.5 Pulse duration characterization

The beams are independently compressed using chirp-scan to a pulse duration of 21 fs FWHM, measured using cross-correlated second harmonic frequency-resolved optical gating (SH-XFROG) in a 10 μm thick BBO crystal placed at the sample position. A typical SH-XFROG trace is shown in Fig.S8.

Table S1: Summary of fit parameters obtained for two distinct spot in the $(PEA)_2SnI_4$

Parameters	Position 1	Position 2
γ_{E1} (meV)	6.0 ±0.3	6.4±0.3
$d\omega_{E1}$ (meV)	9.9 ±0.4	10.2±0.3
γ_{E2} (meV)	6.0 ±0.4	5.3±0.2
$d\omega_{E2}$ (meV)	6.8 ±0.4	11.2±0.3

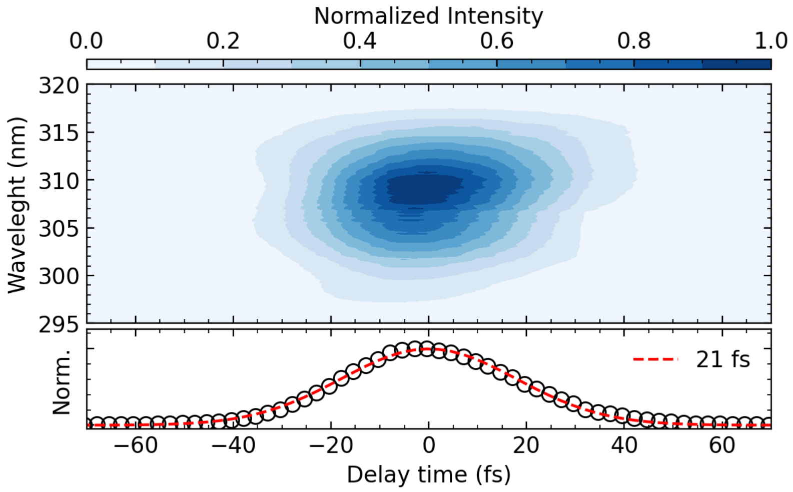

Figure S8: X-Frog characterization of the pulse duration fitted to a Gaussian function.

4 Stochastic scattering model

A complete derivation of the stochastic theory is presented elsewhere [6]–[8]. Here, we summarized the most significant components of the work presented in this letter. The Hamiltonian in equation (S7) describe the scattering events modulated by γ_1, the coupling with a background population, $N(t)$, and V_0 is the exciton self-interaction energy.

$$H_0 = \omega_0 \hat{a}_0^\dagger \hat{a}_0 + \frac{V_0}{2} \hat{a}_0^\dagger \hat{a}_0^\dagger \hat{a}_0 \hat{a}_0 + \gamma_1 \hat{a}_0^\dagger \hat{a}_0 N(t). \tag{S7}$$

The time evolution of the operators is described as

$$\hat{a}_0^\dagger(t) = \exp\left(-i\left(\omega_0 + \frac{V_0}{2}\hat{n}_0\right)t - i\gamma_1 \int_0^t d\tau N(\tau)\right)\hat{a}_0. \tag{S8}$$

The two-dimensional spectra are simulated from response theory. For example, the expressions for a single excitation pathway are,

$$\begin{aligned} R_\alpha(\tau_1,\tau_2,\tau_3) = i^3\mu^4(n_0+1)^2 \exp\left[i(\omega_0 + n_0V_0)\sum_{j=1}^{3}(\pm)_j^{(\alpha)}\tau_j\right] \\ \times \left\langle \exp\left[i\gamma_1 \sum_{j=1}^{3}(\pm)_j^{(\alpha)} \int_0^{\tau_j} dsN(s)\right]\right\rangle, \end{aligned} \tag{S9}$$

$$\left\langle \exp\left[i\gamma_1 \int_0^{\tau_j} N(s)ds\right]\right\rangle \approx e^{i\gamma_1 g_1(t) - \frac{\gamma_1^2}{2} g_2(t)}. \tag{S10}$$

The sign function $(\pm)_j^{(\alpha)}$ takes "+" and "-" depending upon the sign of the photon wavevector entering or leaving the system. $N(t)$ is assumed to be a stochastic variable described by an Ornstein–Uhlenbeck process. The variance of the background population is given by σ and the background relaxation rate is γ. The first and second cumulants are,

$$g_1(t) = \frac{N_0}{\gamma}\left(1 - e^{-\gamma t}\right), \tag{S11}$$

$$g_2(t) = \frac{\sigma^2}{2\gamma^3}\left[2\gamma t + 4e^{-\gamma t} - e^{-2\gamma t} - 3\right] + \frac{\sigma_{N_0}^2}{\gamma^2}\left(1 - e^{-\gamma t}\right)^2. \tag{S12}$$

References

[1] F. Thouin, S. Neutzner, D. Cortecchia, *et al.*, "Stable biexcitons in two-dimensional metal-halide perovskites with strong dynamic lattice disorder," *Phys. Rev. Mater.*, vol. 2, p. 034 001, 3 Mar. 2018. DOI: 10.1103/PhysRevMaterials.2.034001. [Online]. Available: https://link.aps.org/doi/10.1103/PhysRevMaterials.2.034001.

[2] F. Thouin, D. Cortecchia, A. Petrozza, A. R. Srimath Kandada, and C. Silva, "Enhanced screening and spectral diversity in many-body elastic scattering of excitons in two-dimensional hybrid metal-halide perovskites," *Phys. Rev. Res.*, vol. 1, p. 032 032, 3 Dec. 2019. DOI: 10.1103/PhysRevResearch.1.032032.

[3] D. B. Turner, K. W. Stone, K. Gundogdu, and K. A. Nelson, "Invited article: The coherent optical laser beam recombination technique (colbert) spectrometer: Coherent multidimensional spectroscopy made easier," *Rev. Sci. Instrum.*, vol. 82, no. 8, p. 081 301, 2011. DOI: 10.1063/1.3624752. [Online]. Available: https://doi.org/10.1063/1.3624752.

[4] V. Loriot, G. Gitzinger, and N. Forget, "Self-referenced characterization of femtosecond laser pulses by chirp scan," *Opt. Express*, vol. 21, no. 21, pp. 24 879–24 893, Oct. 2013. DOI: 10.1364/OE.21.024879. [Online]. Available: https://opg.optica.org/oe/abstract.cfm?URI=oe-21-21-24879.

[5] M. E. Siemens, G. Moody, H. Li, A. D. Bristow, and S. T. Cundiff, "Resonance lineshapes in two-dimensional fourier transform spectroscopy," *Opt. Express*, vol. 18, no. 17, pp. 17 699–17 708, Sep. 2010. DOI: 10.1364/OE.18.017699. [Online]. Available: https://opg.optica.org/oe/abstract.cfm?URI=oe-18-17-17699.

[6] A. R. Srimath Kandada and C. Silva, "Exciton polarons in two-dimensional hybrid metal-halide perovskites," *J, Phys. Chem. Lett.*, vol. 11, no. 9, pp. 3173–3184, 2020. DOI: 10.1021/acs.jpclett.9b02342.

[7] H. Li, A. R. Srimath Kandada, C. Silva, and E. R. Bittner, "Stochastic scattering theory for excitation-induced dephasing: Comparison to the anderson–kubo lineshape," *J. Chem. Phys.*, vol. 153, no. 15, p. 154 115, 2020. DOI: 10.1063/5.0026467. [Online]. Available: https://doi.org/10.1063/5.0026467.

[8] H. Li, S. A. Shah, E. R. Bittner, A. Piryatinski, and C. Silva-Acuña, "Stochastic exciton-scattering theory of optical line shapes: Renormalized many-body contributions," *J. Chem. Phys.*, vol. 157, no. 5, p. 054 103, 2022. DOI: 10.1063/5.0095575. [Online]. Available: https://doi.org/10.1063/5.0095575.
